# Profiling microbial strains in urban environments using metagenomic sequencing data

**DOI:** 10.1186/s13062-018-0211-z

**Published:** 2018-05-09

**Authors:** Moreno Zolfo, Francesco Asnicar, Paolo Manghi, Edoardo Pasolli, Adrian Tett, Nicola Segata

**Affiliations:** Centre for Integrative Biology, University of Trento, Via Sommarive 9, 38123 Trento, TN Italy

**Keywords:** Metagenomics, Strain-level microbial genomics, Urban microbiome

## Abstract

**Background:**

The microbial communities populating human and natural environments have been extensively characterized with shotgun metagenomics, which provides an in-depth representation of the microbial diversity within a sample. Microbes thriving in urban environments may be crucially important for human health, but have received less attention than those of other environments. Ongoing efforts started to target urban microbiomes at a large scale, but the most recent computational methods to profile these metagenomes have never been applied in this context. It is thus currently unclear whether such methods, that have proven successful at distinguishing even closely related strains in human microbiomes, are also effective in urban settings for tasks such as cultivation-free pathogen detection and microbial surveillance. Here, we aimed at a) testing the currently available metagenomic profiling tools on urban metagenomics; b) characterizing the organisms in urban environment at the resolution of single strain and c) discussing the biological insights that can be inferred from such methods.

**Results:**

We applied three complementary methods on the 1614 metagenomes of the CAMDA 2017 challenge. With MetaMLST we identified 121 known sequence-types from 15 species of clinical relevance. For instance, we identified several *Acinetobacter* strains that were close to the nosocomial opportunistic pathogen *A. nosocomialis*. With StrainPhlAn, a generalized version of the MetaMLST approach, we inferred the phylogenetic structure of *Pseudomonas stutzeri* strains and suggested that the strain-level heterogeneity in environmental samples is higher than in the human microbiome. Finally, we also probed the functional potential of the different strains with PanPhlAn. We further showed that SNV-based and pangenome-based profiling provide complementary information that can be combined to investigate the evolutionary trajectories of microbes and to identify specific genetic determinants of virulence and antibiotic resistances within closely related strains.

**Conclusion:**

We show that strain-level methods developed primarily for the analysis of human microbiomes can be effective for city-associated microbiomes. In fact, (opportunistic) pathogens can be tracked and monitored across many hundreds of urban metagenomes. However, while more effort is needed to profile strains of currently uncharacterized species, this work poses the basis for high-resolution analyses of microbiomes sampled in city and mass transportation environments.

**Reviewers:**

This article was reviewed by Alexandra Bettina Graf, Daniel Huson and Trevor Cickovski.

**Electronic supplementary material:**

The online version of this article (10.1186/s13062-018-0211-z) contains supplementary material, which is available to authorized users.

## Background

Complex communities of bacteria, fungi, viruses and micro-eukaryotes, called microbiomes, are an integral part of human and natural ecosystems [1, 2]. Shotgun metagenomics [3] is a powerful tool to investigate such microbiomes. Indeed, metagenomics has enabled investigations such as those identifying associations between microbial communities and human diseases [1, 4–7] and it has even permitted the discovery of whole new bacterial phyla populating aquatic systems [8]. However, while the microbiomes associated with the human body and with natural environments like soil and oceans have been extensively investigated [2, 9–11], there are instead only a few works characterizing the microbial communities associated with urban environments [12, 13].

The microbial communities populating the urban environment are in direct contact with the city’s inhabitants and their associated microbiomes. Therefore, it is natural to assume there is interplay between the two, with the human inhabitants that have the ability to either acquire or deposit microbes as they travel through urban environments [13–15]. Similarly to the ongoing efforts to characterize the role of microbiomes associated with the built environments (e.g. homes and offices) [16–19] microbial entities thriving within cities should also be considered for their potential interaction with the human microbiome. With the urban population projected to increase by 2.5 billion by 2050 [20–22], it is thus imperative to characterize the microbes that inhabit our cities and their genetic and functional diversity. Indeed, the study of urban microbiomes can be crucial for epidemiology and pathogen surveillance, but also for monitoring the spread of genetic microbial traits like genes responsible for resistance to antibiotics, similarly to what has recently been proposed in clinical settings [23, 24]. Recently, endeavors like the MetaSUB Project have started to characterize the composition of the microbial inhabitants of urban environments [25], but the increasing effort in sampling and metagenomic sequencing from these environments has to be paralleled with either the development or adaptation of computational tools able to fully exploit this urban metagenomic data.

Computational metagenomic approaches for microbiome analysis are in part dependent on the source of the metagenome. The human gut microbiome, for example, can be successfully profiled by assembly-free methods [1] whereas environmental microbiomes characterized by a much larger diversity are typically more dependent on metagenomic assembly [26, 27] and binning [28, 29]. The latest advances in computational metagenomics now permits profiling metagenomes at the sub-species resolution of single strains [30–35] and these methods are particularly suited for the analysis of human microbiomes [36–39]. However, little is known about the utility of existing profiling tools when applied to urban metagenomes, and strain-level analysis has never been applied to the urban setting.

In this work we tested, validated, post-processed and interpreted the application of three strain-level profiling tools originally developed for the human microbiome on a large set of urban metagenomic samples. We analyzed a total of 1614 metagenomes of the MetaSUB dataset distributed as a CAMDA challenge (from now on simply referred to as “MetaSUB dataset”).

## Results and discussion

We applied three strain-level computational profiling approaches for metagenomic data (MetaMLST [35], StrainPhlAn [34], PanPhlAn [33]) to a total of 1614 environmental samples collected across the urban environment of three cities in the United States: New York [13], Boston [12], and Sacramento (unpublished data). The metagenomes were analyzed in the framework of the CAMDA 2017 Challenge conference and are herein referred to as the “MetaSUB data set” which includes the unpublished data of the Sacramento urban environment.

The methods adopted in this analysis have the capability to characterize microbial organisms from metagenomes at the resolution of single strains of known species and they exploit different genomic features, but they have never been applied to urban metagenomes (see Methods).

### Strain typing by multi locus sequence typing using MetaMLST

The first strain typing approach we considered is based on Multi Locus Sequence Typing (MLST). MLST is an effective cultivation-based technique that is frequently used in clinical microbiology and epidemiology to identify and trace microbial pathogens [40, 41]. The method exploits a reduced set of hypervariable loci (usually from 7 to 10) of the target species, which are subjected to Sanger amplicon sequencing and used to define an allelic profile for each strain, called a sequence Type (ST) [42]. MetaMLST [35] is a recent metagenomic cultivation-free extension of the approach that takes advantage of the hundreds of MLST typings available in public databases [43, 44] and performs an *in-silico* MLST analysis on the raw metagenomic reads. MetaMLST detects already observed STs, but can also discover new ones that diverge from the already publicly available types (see Methods).

We applied MetaMLST to profile every species for which an established MLST schema is available. In the MetaSUB dataset a total of 551 samples were positive for at least one species and we recovered a total of 121 known and 510 novel STs of a total of 15 different species (Table 1). The most prevalent species found in the MetaSUB dataset by MetaMLST were *Acinetobacter baumannii*, *Enterobacter cloacae,* and *Stenotrophomonas maltophilia*, and the most prevalent STs were *A. baumannii* ST-71 (detected 20 times) and *Klebsiella oxytoca* ST-44 (detected 8 times).

**Table 1:** Results of MetaMLST applied to the 1614 samples of the MetaSUB dataset. MetaMLST was applied on the full panel of 113 species, detecting in total 121 known and 510 previously unobserved profiles. The table reports the number of samples and STs found for both known and novel STs of the 15 species profiled in the MetaSUB dataset. The prevalence values are normalized over the total number of samples (1614)

Species	Known		Novel			
	Number of samples	Number of STs	Number of samples	Number of STs	Prevalence across dataset	Most prevalent ST (samples)
*Acinetobacter baumannii*	69	22	123	117	11.90%	ST71 (20)
*Enterobacter cloacae*	63	39	89	85	9.42%	ST50 (7)
*Stenotrophomonas maltophilia*	15	12	98	90	7.00%	ST100009 (3)
*Cronobacter* spp*.*	2	2	66	56	4.21%	
*Klebsiella pneumoniae*	13	12	38	38	3.16%	
*Bacillus cereus*	15	11	17	17	1.98%	
*Klebsiella oxytoca*	12	5	18	18	1.86%	ST44 (8)
*Achromobacter* spp*.*	2	2	27	27	1.80%	
*Enrerococcus faecalis*	6	5	18	18	1.49%	
*Propionibacterium acnes*	4	2	18	18	1.36%	
*Escherihia coli*	3	2	16	15	1.18%	
*Pseudomonas fluorescens*	1	1	7	6	0.50%	
*Pseudomonas aeruginosa*	5	1	1	4	0.37%	
*Clostridium botulinum*	1	5	5	1	0.37%	
Total	211	121	541	510		

**Fig. 1:**
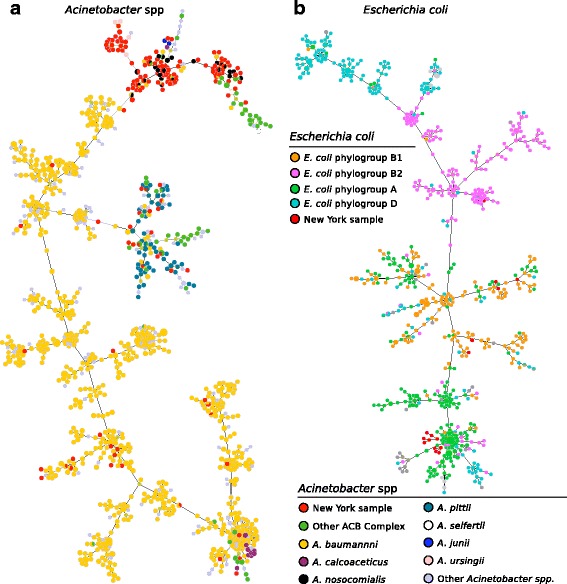
Application of MetaMLST to the 1614 urban metagenomes of the MetaSUB dataset. Minimum spanning trees (MST) were generated on the basis of the allelic profile [86], where each node in the MST represents a Sequence Type (ST) and an edge connects similar STs (i.e. sharing at least one identical locus) with a length proportional to their allelic-profiles similarity. The two MSTs were built with PhyloViz [85]. The 139 detected STs of A. baumannii (**a**) and the 17 STs of E. coli (**b**) are placed in the tree together with the available known STs for which the species is available. In both trees, the STs of the samples from the New York built environment are colored in red

*A. baumannii* was originally described as an environmental bacterium and has been isolated from soil and water [45], but it can also be an opportunistic pathogen [46]. It is one of the six members of the pathogenic group ESKAPE [47] and it is frequently responsible for nosocomial infections. *A. baumannii* and the closely related species *Acinetobacter calcoaceticus*, *Acinetobacter pittii* and *Acinetobacter nosocomialis* are members of the ACB complex [48, 49] and, due to the genetic similarity within this complex, a single MLST schema [50] is used for the whole group [51]. Members of the ACB complex were detected in 192 New York urban metagenomes. When we modelled the detected STs and the reference isolates downloaded from public sources [43, 50] with the minimum spanning tree approach, we found that the majority of the strains from the MetaSUB samples belonged to *A. nosocomialis* and *A. calcoaceticus* STs (Fig. 1a)*.* The majority of the detected STs fall outside the subtree with the known and labelled *A. baumannii* STs. Overall, this demonstrates the presence of *Acinetobacter* and therefore potentially opportunistic pathogens in the urban environment and highlights how a very well defined subtree of the group comprises strains that are found in the ecological niche of the urban environment.

We next focused on *Escherichia coli*, a common member of the human gut microbiome that is also found in the environment. *E. coli* has a large number of sequence types that can be classified in phylogroups, with the majority of commensal strains found within the phylogroups A and B1 [52, 53], and opportunistic pathogenic strains, such as ExPEC *E. coli*, falling in phylogroup B2 [54]. MetaMLST detected *E. coli* in 19 New York subway samples and by comparing the recovered STs with the references available in BigsDB [43], we were able to assign the strains to the *E. coli* phylogroups **(**Fig. 1b**)**. The majority (53%) of the samples fall in the mainly non-harmful phylogroup A. One sample harboured a novel *E. coli* type (*adk 37; fumC 38; gyrB 19; icd 37; mdh NEW; purA 11; recA 26*) very closely related to ST-95 (3 SNVs over 3423 total nucleotides) profile, which is one of the most commonly found *E. coli* phylogroup B2 strains [55, 56]. These results highlight that MetaMLST is capable of detecting microbes at the strain level in complex environmental communities, thus enabling epidemiology modelling from urban samples.

### Phylogenetic strain characterization using extended single nucleotide variant profiling

MetaMLST is a rapid method for the strain level profiling of a species for which a MLST schema exists and strains are identified by exploiting single nucleotide variants (SNVs) within a small set of genetic loci. With the goal of extending this approach, we recently developed StrainPhlAn [34], which characterizes strains in metagenomes by targeting the SNVs within clade-specific markers (> 200 markers for each species). The increased number of loci enables a finer resolution for distinguishing closely related strains, and unlike MetaMLST is applicable to any species of interest for which at least one reference genome is available.

We applied StrainPhlAn to the microbial species identified in the MetaSUB dataset by the species profiling tool MetaPhlAn2 [57]. In total, we identified 539 microbial species with a relative abundance above 0.5%. Of these, 155 were present in more than 10 samples with only a minor correlation between the sequencing depth of each sample and the observed number of species (Additional file 1: Figure S1). In samples from New York we found *Pseudomonas stutzeri and Stenotrophomonas maltophilia* to be the most abundant carachterized species (Additional file 2: Table S1). Boston was instead dominated by *Propionibacterium acnes* as previously reported [12]*,* while the city of Sacramento showed a high prevalence of species in the *Geodermatophilaceae* family and *Hymenobacter* genus, which are known environmental bacteria [58, 59]. In addition, in the Sacramento samples we found other potential opportunistic pathogens such as *Halomonas* spp. [60] and *Kocuria* spp., which is a species commonly found both in soil and human skin [61–63].

**Fig. 2:**
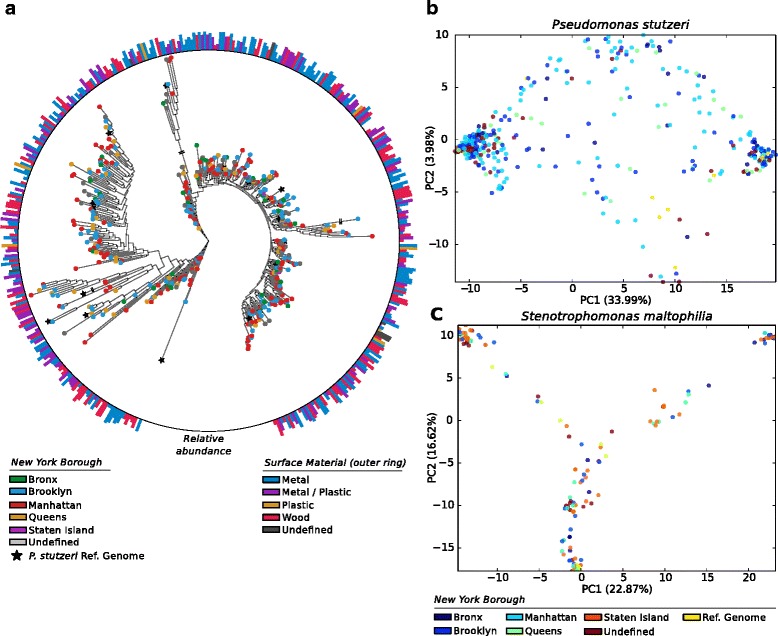
Strain-level phylogenetic analysis of the two most prevalent bacterial species identified in the metagenomic samples of the New York urban environment. The phylogenetic trees are inferred by applying StrainPhlAn on the raw sequencing reads. **a** Maximum likelihood phylogeny of *P. stutzeri* (built with RAxML [83] internally in StrainPhlAn). The root of the phylogenetic tree is placed using *P. putida* as an outgroup. Nodes are colored by the New York Borough from which the sample was collected, with black stars representing reference genomes. The height of the bars of the bar-plot on the outer ring represents the relative abundance of *P. stutzeri* as computed by MetaPhlAn2, while the color represents the surface material of the sample. The lengths of branches marked with a double horizontal line are reduced by 50% (**b**, **c**) PCA plot based on the genetic distance computed on the species-specific markers sequences of 416 samples and 18 reference genomes of *P. stutzeri* (**b**) and 111 samples and 80 reference genomes of *S. maltophilia* (**c**). The points are colored according to the New York Borough

The most prevalent species identified in New York, *P. stutzeri,* was identified in 967 samples across the New York dataset. Of those, 416 samples harboured *P. stutzeri* at a sufficient coverage to be profiled by StrainPhlAn. The StrainPhlAn inferred phylogeny highlighted the presence of three clusters of *P. stutzeri* strains that do not correlate with the geographic area from which the sample was taken (Fig. 2a) nor are they correlated with other sample characteristics such as surface material (Fig. 2b). This may suggest that samples collected in high-density and high-transit urban environments may be extremely heterogeneous without evidence of sub-niche selection. Alternatively, this could be a reflection of these species being carried around between stations and other surfaces of the urban furniture by commuters. Although this has never been previously observed, further research is needed to demonstrate such kind of events.

We next profiled *S. maltophilia*, which is the second most prevalent species in the New York dataset. *S. maltophilia* is not only a common environmental bacterium, but also a nosocomial opportunistic pathogen in immunocompromised patients [64]. We found 654 samples in which *S. maltophilia* was present. Of those, 111 samples harboured *S. maltophilia* at a sufficient coverage to be profiled by StrainPhlAn and were considered in the phylogenetic analysis. From the ordination plot based on inter-strain genetic distances, we identified three main clusters (Fig. 2c) that, similarly to *P. stutzeri*, did not show any correlation with either the geography or the surface material from which the sample was taken, supporting the hypothesis that the genetic structures of microbial species and sample characteristics in urban environments tend to be uncoupled.

### Evidence for high intra-species strain heterogeneity in urban microbiome samples

**Fig. 3:**
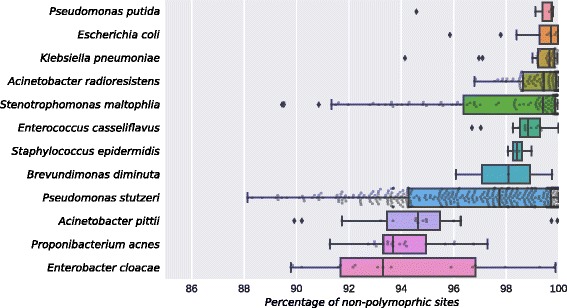
Strain heterogeneity distribution for a set of highly prevalent species across the MetaSUB dataset. For each species, we report the distribution of the average rate of non polymorphic sites in the sample (see Methods). The boxes show the first and third quartiles of the dataset, the bar inside the box represents the median (second quartile), while the whiskers extend to cover the 99.3% of the distribution. External points represent outliers

Complex microbial communities can harbor multiple strains of the same species. This is a well-known characteristic for both human associated [34, 65] and environmental microbiomes, but profiling multiple related strains simultaneously within the same sample is currently very challenging [3]. It is nonetheless important to quantify the strain level heterogeneity within a sample. Similarly to what we did previously for the human gut microbiome [34], we investigated the strain heterogeneity for the species in the urban microbiomes. This was performed by quantifying the rate of polymorphic nucleotides for each position along of the species’ reads-to-markers alignments (see Methods). We computed the estimate of strain-heterogeneity for a number of the most prevalent species in each city (Fig. 3).

We observed a higher intra-species variability in the MetaSUB dataset than what we previously found in the human gut microbiome [34], thus suggesting that the higher complexity and species richness of environmental microbiomes [3] is also reflected at the sub-species level. For instance, *E. cloacae* and *P. acnes* show high median polymorphic rates (Fig. 3) suggesting that more than one strain of the species is present within the sample. In contrast, for *P. putida* and *E. coli* a single strain dominates the community for most of the samples. We also highlight the presence of species characterized by higher polymorphic-rates inter quantile ranges (IQR), like *P. stutzeri* and *S. maltophilia*, suggesting that these species are sometimes single-strain dominated and other times they are represented simultaneously by many distinct strains. We can speculate that the higher percentages of polymorphic rates can be due to the high number of distinct microbial sources (subway users) coming in contact with the sampled surfaces. Overall, these results highlight that the same species can harbour a substantial strain heterogeneity across samples, and that these strains can sometimes coexist in the same niche.

### Functional profiling of strains based on species’ pangenomes

MetaMLST and StrainPhlAn are based on the comparison of the SNVs within species-specific markers. Microbial species can also be profiled according to the presence or absence of their gene repertoire [66–68]. In order to profile strains according to their genomic content (gene repertoires), we applied PanPhlAn, a software tool that outputs the gene presence-absence profile for a given species in a metagenome. In addition to the inference of the relatedness of strains, this approach can also be useful to identify specific strain-specific genomic traits. These include, for instance, antibiotic resistance and virulence determinants that can be present in only a subset of the strains in a species. In previous studies, PanPhlAn proved successful in detecting pathogenic species besides commensal strains of *E. coli* [33, 69], but again this was performed only in human-associated microbiomes.

**Fig. 4:**
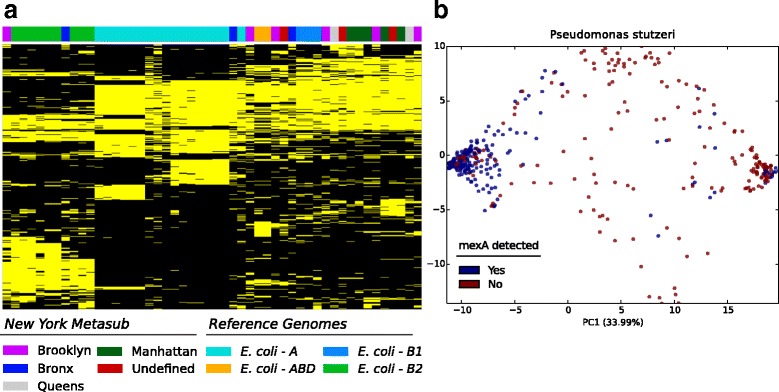
Functional profiling of the species of the MetaSUB dataset across the New York urban environment. **a** PanPhlAn presence-absence matrix of *Escherichia coli*. The rows represent the gene families while columns represent the samples. The top colorbar highlights the New York Borough and the *E. coli* reference genomes’ phylogroups. In the heatmap yellow corresponds to presence, black corresponds to absence. Only the gene-families present in less than 90% and more than 10% of the samples were included. **b** PCA plot based on the genetic distance computed on the species-specific markers sequences of 416 samples and 18 reference genomes of *Pseudomonas* *stutzeri* as reported in Fig. 2c. Each point is a sample and is colored according to the presence-absence of the *mexA* component of the Pseudomonas MexAB-OprM efflux system

To test whether differences in strains could be observed in the urban metagenomes, we applied PanPhlAn to target *E. coli* in the New York dataset. *E. coli* was detected at sufficient coverage for profiling in 19 samples, of which five were among those profiled with MetaMLST. Comparing the presence-absence profiles of this 19 *E. coli* with a selection of reference genomes (i.e. those contained in PanPhlAn), revealed that the New York samples had a genetic functional potential similar to the largely non-pathogenic phylogroups A and B1, similarly to what was shown with MetaMLST. Conversely, only two samples were close to phylogroup B2 (Fig. 4a).

An analysis based on the genomic content of the species of interest can highlight the presence of specific traits of a species within a complex microbial community [70]. For example, it would be useful for epidemiological and microbial surveillance to profile and trace directly specific antibiotic resistance genes or virulence factors. To test whether the identification of a specific genetic capability could be achieved in the urban environment, we applied PanPhlAn to profile a species commonly identified in the MetaSUB dataset, *P. stutzeri*, which is also known to encode for different antibiotic resistances [71, 72]. As an example, we specifically targeted the presence of the *mexA* gene, a component of the MexAB-OprM efflux system, which can confer resistance to numerous antibiotics and other antimicrobial agents [73, 74]. We found that *P. stutzeri mexA* strains were present in a subset of the New York samples. In total, 372 New York samples encoded *mexA*, while 56 samples did not (Fig. 4b), and the PanPhlAn results were generally in agreement with the three clusters model obtained with StrainPhlAn. Interestingly, while clusters of *P. stutzeri* grouped both according to the genetics and the presence/absence of *mexA*, few strains that contained *mexA* clustered genetically with strains that did not contain the gene and vice-versa. Indeed, the presence of the same protein encoded by two strains that are genetically very distant may imply that the presence of *mexA* in some of these strains is imputable to some degree of lateral gene-transfer.

Overall, these findings highlight that it is possible to type at the functional level populations in the urban metagenomes using strain-level approaches based on the overall genomic repertoire and that samples can be investigated at a deeper level to unravel the diversity of specific microbial genetic traits among complex communities.

### Comparing strain profiling by SNVs and gene content.

The two approaches we presented so far can reflect the strain-level diversity within a species, either taking in consideration the genomic content of strains, or their phylogenies. However, the two methods can convey different information. For example, as highlighted above for the *mexA* gene in *Pseudomonas stutzeri*, two strains could be phylogenetically very similar while displaying different resistance capabilities, which is why these methods should be considered complementary. In order to further evaluate the consistency and complementarity of the two approaches to profile strains, we performed a comparison between the two distance measures of PanPhlAn and StrainPhlAn. We investigated a panel of the urban species already analyzed above, and computed the pairwise phylogenetic (StrainPhlAn) and phylogenomic (PanPhlAn) distances within the samples (see Methods).

**Fig. 5:**
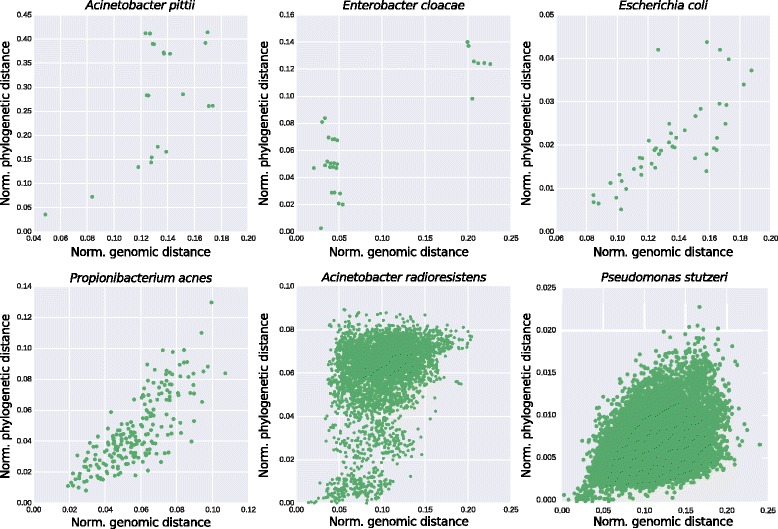
Normalised phylogenetic distance vs genomic-content distance within samples of six representative species of the MetaSub dataset. Each data point refers to a pair of two strains of the same species in different samples. The genomic distance is defined as the normalised Hamming distance between binary vectors of presence-absence as reported by PanPhlAn. The phylogenetic distance is defined as the branch length distance of the two leaves in the StrainPhlAn phylogenetic tree, normalised over the total branch length of the tree. Pearson’s correlation coefficients are *A. pittii*: 0.57, *E. cloacae*: 0.85, *E. coli*: 0.75, *P. acnes*: 0.79, *A. radioresistens*: 0.34 and *P. stutzeri*: 0.41. *P*-values are always lower than 1e-5

We found that genetic and genomic variations within the same sample are generally correlated for all the six species considered, confirming that both measures are an effective proxy for strain relatedness and identity across samples (Fig. 5). However, the correlation coefficient varied across species, spacing from 0.34 (*p*-value 5.2e^− 219^) for *A. radioresistens* to 0.85 (p-value 6.9e^− 17^) for *E. cloacae.* These values reflect a different consistency between the phylogenetic signal and the evolutionary modifications of the functional profiles.

We also highlight the presence of samples that, regardless of the species, are much more functionally similar than the phylogenetic modeling would suggest, possibly reflecting convergent functional adaptation. Conversely, increased genomic content distances, suggests rapid functional divergence potentially due to plasmids, bacteriophages, or other lateral gene-transfer events. Such patterns, detected for example in *P. stutzeri* and *A. radioresistens*, are suggesting that strains can be very similar according to phylogeny and still be notably diverse in their functional potential.

## Conclusions

We presented here the application of three strain level profiling tools to environmental urban metagenomics. While these tools were specifically developed for the context of the human microbiome, we highlighted that it is possible to apply them to efficiently perform strain profiling in the context of the urban environment. We provide evidence that potential pathogenic species can be recovered, typed, and traced across microbial communities that are wider and more complex than the ones we observe in the human microbiome. Moreover, the phylogenetic relation of strains in the same species and their functional repertoires can be simultaneously profiled, thus providing a more complete characterization of strains in the samples. These findings suggest that the tools presented above are effective for the purposes of pathogen surveillance and epidemiology in the context of environmental metagenomics.

The three methods presented in this work are capable of profiling microbes that are close to a reference sequences (MetaMLST), or for which a sequenced genome for the target species exists (StrainPhlAn and PanPhlAn). Because environmental microbiomes can contain a larger amount of unknown species [3] compared to human associated microbiomes, this dependency on already sequenced data can limit strain profiling to only a portion of the whole microbiomes. Additional profiling approaches can exploit metagenomically assembled contigs or genomes [3, 26–28, 30, 75] which are widely employed in environmental metagenomics and are necessary when targeting the fraction of not previously sequenced taxa. Our strain-profiling methods can be extended to use metagenomic assembled genomes as reference, and this would provide a combined assembly-based and assembly-free tool to explore the uncharacterized diversity in microbiome samples with strain-level resolution.

This work demonstrates that assembly-free strain-level profiling through SNVs and genomic content is a promising technique for comprehensive strain-resolved metagenomics in the urban environment.

## Methods

We profiled a total of 1614 samples with three strain-level profiling tools described below. The dataset comprehended 1572 samples collected in the city of New York (NY, U.S.A., [13]), 24 samples collected in the city of Boston (MA, U.S.A., [12]) and 18 samples collected in the city of Sacramento (CA, U.S.A., unpublished).

Samples from Boston and New York are publicly available at NCBI under accession numbers PRJNA301589 and PRJNA271013, respectively.

### MetaMLST

MetaMLST [35] is a tool for strain-level typing and identification from metagenomic data. It exploits the Multi Locus Sequence Typing (MLST) approach and performs an *in-silico* reconstruction of the MLST loci using a reference-guided majority rule consensus method. MetaMLST detects the sequence type (ST) of the most abundant strain the target species in the sample. Specifically, MetaMLST reconstructs the sequence of each locus from the raw metagenomic reads and compares it with a database of previously observed variants. Additionally, MetaMLST is capable of identifying new loci that diverge from the closest known sequence by up to 10 single nucleotide variants (SNVs). Hence, MetaMLST detects both known and novel (i.e. previously unobserved types) STs.

We applied MetaMLST version 1.1 to the entire MetaSUB dataset by mapping the raw reads against the MetaMLST database as of April 2017, consisting of 113 organisms, 798 loci, 46.2 Mbp and 12,929 total profiles. The mapping was performed with bowtie2 [76], version 2.2.6 as previously described (parameters: -D 20 -R 3 -N 0 -L 20 -i S,1,0.50 -a --no-unal) [35]. Alignment files were sorted with Samtools version 1.3.1 [77]. We reported only the species for which at least one known ST could be detected.

### StrainPhlAn

StrainPhlAn [34] is a tool for identifying the specific strain of a given species within a metagenome. The tool is designed to track strains across large collections of samples and takes as input the raw metagenomic reads in FASTQ format. After mapping the reads against the set of species specific markers (> 200 per species), StrainPhlAn reconstructs the sample specific marker loci using a variant calling approach and outputs the sequences of each sample-specific marker in FASTA format. Sequences are extracted from the raw reads using a reference-free majority rule that filters out noisy regions. The resulting sequences were then concatenated and aligned by StrainPhlAn with Muscle version 3.8 [78]. In this work, we applied StrainPhlAn to the whole MetaSUB dataset and investigated a panel of 12 species that were locally prevalent in the three cities of the MetaSUB dataset. The reconstructed markers were used to build the phylogenetic tree and the PCA plots of *P. stutzeri* and *S. maltophilia* (Fig. 2). The reads-to-markers alignments of the 12 species were used in the calculation of the polymorphic rate (Fig. 3). StrainPhlAn version 1.0 was used with default parameters, using the mpa_v20_m200 markers database of MetaPhlAn2 [57]. The mapping against the markers was performed with Bowtie2, version 2.2.6, with the parameters implemented in the StrainPhlAn pipeline [34].

### PanPhlAn

Pangenome-based Phylogenomic Analysis (PanPhlAn) [33] is a strain-level metagenomic profiling tool for identifying the gene composition of a strain of a given species within metagenomic samples. The approach of PanPhlAn is based on the identification of presence/absence patterns in the genomic content within the members of the same species, across complex metagenomic samples. As the pre-built PanPhlAn database did not include the pangenome of *Pseudomonas stutzeri*, we built a custom db from 19 high-quality reference genomes (NCBI accession numbers: ASM19510v1, ASM21960v1, ASM26754v1, ASM27916v1, ASM28055v1, ASM28295v1, PseStu2.0, ASM32706v1, PstNF13_1.0, PstB1SMN1_1.0, ASM59047v1, ASM66191v1, ASM95268v1, ASM98286v1, ASM103864v1, ASM106422v1, ASM127647v1, ASM157508v1) which were first annotated using Prokka [79] and then clustered into gene-families with Roary [80]. We profiled the 1572 New York samples from the MetaSUB dataset with PanPhlAn version 1.2.1.3.

### Visualization and statistical tools and phylogenetic distances

We defined the phylogenomic distance between two samples as the pairwise Hamming Distance on the PanPhlAn presence-absence profile for each sample, represented as binary vectors where 1 represents the presence of the gene, and 0 represents its absence. The phylogenetic distance was calculated as the minimal total branch-length distance between leaf nodes, normalized by the total branch length, using custom python scripts based on BioPython BaseTree [81, 82].

The phylogenetic trees were built with RAxML [83] version 8.1.15 (parameters: -p 1989 -m GTRCAT) and plotted with GraPhlAn [84]. Minimum Spanning Trees were drawn with PHYLOViZ 2 [85] using the goeBURST Full MST algorithm [86]. The principal component analysis (PCA) plots were drawn with the scikit-learn package using the aligned concatenated markers sequences of StrainPhlAn as arrays of binary features. All the overlaid metadata used to colorize the trees and PCA plots came from the respective studies.

The presence of polymorphic sites within the reads-to-markers alignment was calculated and reported with StrainPhlAn [34], testing the non-polymorphic null hypothesis on a binomial test on the nucleotides distribution of each position in the alignment. The plots were drawn with python packages seaborn and matplotlib [87].

## Reviewers’ comments

### Reviewer’s report 1 - Alexandra Bettina Graf, FH campus Wien

**Reviewer comments:** The authors use three different tools, MetaMLST, StrainPhlAn and PanPhlAn to profile three urban metagenome datasets (New York, Boston, Sacramento – as yet unpublished), which were presented as one of the CAMDA challenges. Their stated goals are the characterization of organisms in urban environments at single strain level and the discussion of inferable biological insights from the analysis. Although all three tools were already published by the authors and already tested in with dataset from the human microbiome, the application of the method for urban metagenome data is interesting for two reasons: (1) urban microbiomes are generally more complex than the human microbiome and (2) urban microbiomes are in close contact with the human population within cities. Reaching sub-species and strain level resolution is of great advantage in relation to determining the pathogenicity of organisms, and is still not a trivial task for complex datasets. The authors show that the presented approach can be used to investigate urban metagenome samples on a sub-species and strain level and that the results can be used to further investigate the specific dynamics of the microbial communities found in urban environments. The authors further show that the analysis of the pathogenic potential and dynamics of urban metagenome samples can result in valuable information in the context epidemiological models (AMR evolution, AMR dynamics – lateral gene transfer, and mobility) and surveillance of pathogens. The described methods can only be applied to the subset of the sample for which reference data is available. This proportion is, in the case of the urban microbiome, much smaller than for the human microbiome. The authors correctly recognize this limitation in their work. Despite this limitation, I believe the authors have made a valuable contribution to the field. Minor recommendation: It would be interesting to hint on any effect the data quality and coverage could have on the results, since these factors can significantly influence the observed species (strain) diversity. Was there a difference in diversity seen between the different datasets? Did this influence the results?

Author’s response: *We thank the reviewer for her assessment, with which we agree. To better clarify on the impact of the coverage on the detection capabilities of the methods we used, we compared the number of detected species against the read count of each profiled sample and reported the results in the* Additional file 1: Figure S1*. We discussed the results in the text in the Results section. We also corrected all the minor issues pointed out by the reviewer.*

### Reviewer’s report 2 - Daniel Huson, University of Tübingen

**Reviewer comments:** Summary**:** This paper applies three methods, MetaMLST, StrainPhlAn and PanPhlAn to 1614 metagenomic sequencing samples obtained from the urban environment in NYC, Boston and Sacramento. It addresses the question how well these methods perform on such data, given that they were originally developed for the analysis of human-body-associated samples. The authors demonstrate that the methods are indeed applicable and can provide information on strains present in the samples. I think that this is a worthwhile analysis and provides a good showcase for the use of the discussed methods. Recommendations: In the introduction you make some strong statements about the role of the urban environment and the interplay between the microbiomes of humans and the urban environment. You mention pathogen surveillance and the spread of antibiotics. It would be very useful to provide some references for these statements, or to formulate them more tentatively. While it seems very plausible to me that microbes may jump off and jump on humans at subway stations, I don’t know whether this has been conclusively shown. Also, “Urban environments, despite being important for human health,…” requires the citation of a paper showing this. So, in general, I recommend that you distinguish very precisely between what has been shown and what is speculation when discussing the role of the urban environment in human health. In the Methods section, you provide a short summary of the MetaMLST method. From this description, I don’t understand how MetaMLST addresses the combinatorial problem of matching different locus types with each other? E.g., if there are 7 loci and for each we find 10 types, then there are 10^7 different possible STs. It would be helpful (for me at least) if you could add a couple of sentences explicitly explaining how this issue is addressed.

Author’s response: *We thank the reviewer for his comments and we do agree that the introduction needed to be partly amended to better address the relationship between environmental microbes and human microbiome. Although we could not cite references in the abstract due to Biology Direct’s authors guidelines, we added two references in the relevant sections of the introduction. Additionally, we better clarified on the importance of microbiomes in the built environment by editing accordingly the second paragraph of the introduction. To our knowledge, transfer of human microbes between transportation lines and stations has not been described before in literature: we now mention it in the manuscript. We further amended the text by better explaining the analysis performed by MetaMLST, and by highlighting that only the most abundant variant of each target species is reported by the tool. We also corrected all the minor issues pointed out by the reviewer.*

### Reviewer’s report 3 - Trevor Cickovski, Florida International University

**Reviewer comments:** The article provides a quite thorough analysis of urban environments using several analysis tools that have been used primarily to study the human microbiome, and presents several very interesting and sometimes encouraging findings; especially with respect to finding more of a difference in microbiomes between cities compared to areas within the same city, being able to detect and profile pathogenic bacteria, and supporting the growing necessity of subspecies-level profiling. While there is no methodological novelty, I do very much like the creative combination of existing packages in a way that can thoroughly analyze an underexplored domain in this field. I believe that is often just as important and viewing the purposes of Biology Direct, discovery and application notes as well as reviews are perfectly acceptable. The paper is well-written and organized well, I was clear of the goals, how each portion contributed to those goals, what was found and where it was going. I therefore recommend the paper to be published as is.

Author’s response: *We thank the reviewer for his comment on the manuscript.*

## Additional files

Additional file 1: **Figure S1.** Scatterplot contrasting for each sample the number of successfully profiled species against the metagenome size (in million reads). Each dot corresponds to a sample in the MetaSUB dataset. The number of detected species was calculated with MetaPhlAn2 by requiring a species to have a relative abundance higher than 0.5% within the sample. (PDF 113 kb)

Additional file 2: **Table S1.** MetaPhlAn2 output table on the whole MetaSub dataset. Values represent the relative abundances detected for each sample (in the columns). (CSV 16816 kb)

## Abbreviations

IQR: Inter Quantile Range
MLST: Multi Locus Sequence Typing
MST: Minimum Spanning tree
PCA: Principal Component Analysis
SNV: Single nucleotide variant
ST: Sequence Type
