# Supplementary figures and images for: Profiling microbial strains in urban environments using metagenomic sequencing data
Source: Biol Direct. 2018 May 9;13:9. doi: 10.1186/s13062-018-0211-z (PMC5944035; doi:10.1186/s13062-018-0211-z)

Number of species (>0.5% rel. ab.)

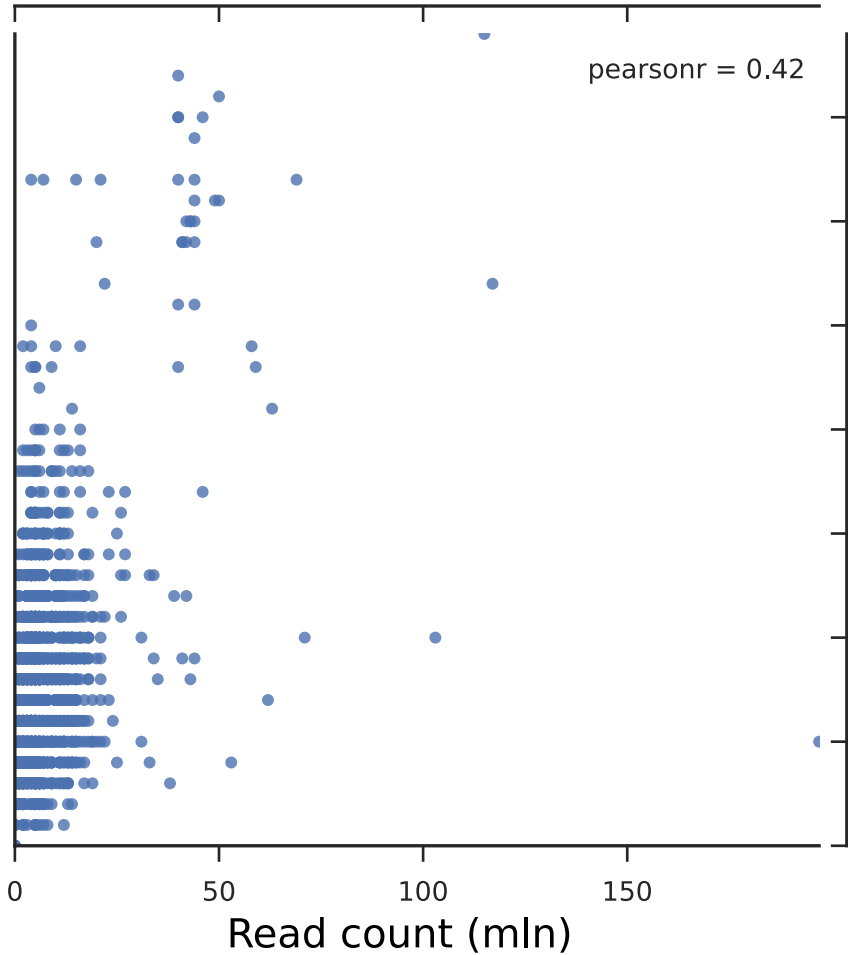

Supplement: Supplementary file 1 — Figure S1. Scatterplot contrasting for each sample the number of successfully profiled species against the metagenome size (in million reads). Each dot corresponds to a sample in the MetaSUB dataset. The number of detected species was calculated with MetaPhlAn2 by requiring a species to have a relative abundance higher than 0.5% within the sample. (PDF 113 kb) [file 13062_2018_211_MOESM1_ESM.pdf]
